# Epidemiological profiles of recurrent malaria episodes in an endemic area along the Thailand-Myanmar border: a prospective cohort study

**DOI:** 10.1186/s12936-019-2763-5

**Published:** 2019-04-08

**Authors:** Saranath Lawpoolsri, Jetsumon Sattabongkot, Jeeraphat Sirichaisinthop, Liwang Cui, Kirakorn Kiattibutr, Nattawan Rachaphaew, Kritsana Suk-uam, Amnat Khamsiriwatchara, Jaranit Kaewkungwal

**Affiliations:** 1 Center of Excellence in Biomedical and Public Health Informatics (BIOPHICS), Faculty of Tropical Medicine, Mahidol University, Bangkok, Thailand; 2 Department of Tropical Hygiene, Faculty of Tropical Medicine, Mahidol University, Bangkok, Thailand; 3 Mahidol Vivax Research Unit, Faculty of Tropical Medicine, Mahidol University, Bangkok, Thailand; 4 Bureau of Vector-Borne Diseases, Department of Disease Control, Ministry of Public Health, Nonthaburi, Thailand; 5 Division of Infectious Diseases and Internal Medicine, Department of Internal Medicine, University of South Florida, Tampa, USA; 6 Vector-Borne Disease Control Center 2.3, Ministry of Public Health, Tak, Thailand

**Keywords:** Recurrent malaria, Thai-Myanmar border, Anti-malarial drug resistance, Multiple episodes, Low malaria transmission, Malaria elimination

## Abstract

**Background:**

In low malaria transmission areas, many people acquire multiple malaria infections within a single season. This study aimed to describe the pattern and epidemiological profile of malaria recurrence in a hypoendemic area of western Thailand and identify factors associated with having multiple malaria episodes.

**Methods:**

An open cohort of 7000 residents in seven clusters along the Thai-Myanmar border was followed during a 6.5-year period (2011–mid 2017). Symptomatic and asymptomatic malaria infections were detected by passive case detection (PCD), weekly household visit, and mass blood surveys every 4–6 months. Malaria recurrence was defined as subsequent parasitaemic episodes occurred later than 7 days after receiving anti-malarial treatment. This study focused on analysis of recurrent episodes that occurred within 1 year after treatment. Numbers of malaria cases with single and multiple episodes were compared between clusters. Kaplan–Meier curve was performed to determine the intervals of recurrent episodes by *Plasmodium* species and age groups. The ordinal logistic model was used to determine factors associated with multiple malaria episodes, and to compare with single episodes, and those with no malaria infection.

**Results:**

The cumulative incidence of malaria in the study area was 5.2% over the 6.5 years. Overall, 410 malaria patients were detected. Of these patients, 20% and 16% had multiple malaria episodes during the entire period and within 1 year after initial treatment, respectively. About 80% of repeated malaria episodes were caused by the same *Plasmodium* species as the primary infections. The median interval and interquartile range (IQR) between the first and second episode was 88 (43–175) days for all parasites, 56 (35–133) days for two *Plasmodium falciparum* episodes, and 90 (59–204) days for two *Plasmodium vivax* episodes. The interval between the episodes was increased with age. Factors significantly associated with multiple episodes of malaria infection included male sex, young age, Karen ethnicity, forest-related occupation, and having other malaria infected persons in the same house in the same period.

**Conclusions:**

People who have multiple malaria episodes may play an important role in maintaining malaria transmission in the area. Understanding epidemiological profiles of this group is important for planning strategies to achieve the elimination goal.

## Background

People living in malaria endemic areas may experience more than one malaria attack even in a single season. The distribution and determinants of the multiple malaria attacks depend on the local epidemiological settings. In the hyperendemic areas of Africa, children may suffer repeated *Plasmodium falciparum* attacks every 4 to 6 weeks over many years [1–3]. Even in low-transmission settings in Africa, it was estimated that on average a person might have 1–3 episodes of malaria infection in a year [4]. A study conducted in the Thai-Myanmar border region of Southeast Asia, where both *P. falciparum* and *Plasmodium vivax* are symptomatic, found that the cumulative proportions of patients having recurrent *P. falciparum* and *P. vivax* infections within 63 days after treatment of acute *P. falciparum* malaria were 21.5% and 31.5%, respectively [5].

Several terms are used to define the repeated episodes of malaria in a patient. Recurrence of parasitaemia after treatment can result from: (a) recrudescence from asexual parasitaemia, (b) relapse from hypnozoites, and (c) reinfection by a new mosquito inoculation [6–8]. Recrudescence occurs with all *Plasmodium* species when the blood-stage parasites are not completely eradicated and subsequently re-expanded in number after drug concentrations in the blood decline. Relapse occurs mostly in *P. vivax* and *Plasmodium ovale* when parasitaemia and clinical manifestations reappear due to re-activation of dormant hypnozoites in the liver. Reinfection is an infection acquired from a new mosquito bite. Although secondary infection is usually genetically different from that of the primary infection, it is difficult to distinguish precisely between relapse, recrudescence and reinfection in the case of *P. vivax* infection [6–9].

Recurrence after treatment leads to a new clinical episode with a risk of complications for the patient [10]. Compared to the life-threatening *P. falciparum*, *P. vivax* is seemingly much less virulent, thus the term benign tertian, but there are studies showing that recurrent patients with this parasite could become difficult to treat and sometimes fatally ill [11]. *Plasmodium vivax* infection may result in a decrease in peripheral blood lymphocyte subsets, and the number of recurrent episodes was found to be correlated with an unbalanced and exacerbated immune response and unbalanced ratio of the CD4+/CD8+ T-cells [12]. Malaria recurrence not only carries biological or clinical consequences, but also affects socio-behavioural aspects. Previous studies in Thailand and Sri Lanka reported the association of repeated malarial infections with poor school performance of children in language and mathematics [13, 14]. Thus, it is critical to design more effective intervention programmes targeting these recurrent cases, which often requires deep knowledge about the epidemiological patterns of recurrent malaria infections [15]. The strategy of early diagnosis and treatment, combined with vector control and human behavioural change, has been shown to be able to partially reduce malaria incidence in the population living on the Thai-Myanmar border [16, 17].

The determinants of malaria infection can be observed at different levels: (a) individual level including biological and disease-related factors; (b) household and community levels covering social and economic factors, and (c) environmental and institutional factors [18–20]. At the individual level within the human host the determinants include demographic, biological, genetic, immunological, and pathophysiological mechanisms that predispose people to disease. At the household and community levels there are strong links between malaria incidence with socio-economic, occupation-related factors as well as the pattern of treatment-seeking behaviour and access to healthcare and services in the community settings. Environmental and institutional factors that are found associated with malaria incidence include not only the climate and spatial locations but also the political and legal conflicts in the endemic area such as migration, displacement, and cross-border situations [20].

Countries in the Greater Mekong Subregion (GMS) are moving toward regional malaria elimination. In the GMS, malaria distribution is highly heterogeneous on both large and small geographical scales. On a regional scale, an upsurge in malaria incidence has been observed in Cambodia in 2017 [21], despite that malaria incidence continues to decline in the entire GMS. On a country-wise scale, malaria cases with both single and multiple episodes still linger on in remote areas along international borders. It is thus essential to understand the changing malaria epidemiology in order to guide the national malaria control programmes (NMCPs). This study aimed to identify the magnitude and risk factors of malaria cases with repeated episodes at the individual and household levels and to determine the time to subsequent episodes of malaria. This study utilized longitudinal study methods of a cohort-based demographic surveillance system and repeated cross-sectional mass blood surveys (MBS).

## Methods

### Study design and study population

This study was part of The International Center of Excellence for Malaria Research (ICEMR) project that was established to evaluate the impact of malaria control interventions across endemic regions that differ in the dominant *Plasmodium* species, mosquito vector species, and human populations. One goal of the ICEMR programmes is to generate basic epidemiological information across a range of epidemiological settings [22]. This study combined field activities of the Southeast Asian ICEMR including health facility-based surveillance, cohort study, and cross-sectional surveys.

The study was conducted in seven clusters (subsets of 7 villages) of an open cohort for 6.5 years (2011-mid 2017) following approximately 7000 people in Tha Song Yang District, Tak Province where malaria incidence has been persistent for decades. The seven clusters varied in population sizes representing semi-rural, rural, and remote areas along the Thai-Myanmar border. The study employed both passive case detection (PCD) and active case detection (ACD). For PCD, symptomatic malaria cases were detected at hospitals, clinics and malaria posts, while ACD was performed during weekly home visits to collect data on symptomatic and asymptomatic cases as well as population movement within the cohort. In addition, MBS in the cohort also was performed three times a year during 2011–2015 with the aim to detect both hidden symptomatic and asymptomatic cases.

At the beginning of the project, household visits by field workers and/or trained community health volunteers were performed. Informed consent was obtained from all study participants in each cluster of the cohort. A unique study identification number was assigned to an individual in each cluster who agreed to participate. Demographic variables included gender, age, occupation, education level, ethnicity and nationality. Households were also given house codes along with their spatial coordinates. As an open cohort, besides the fixed numbers of member in the initial cohort, there might be residents who moved out or new comers who moved in the cluster. New members at each household visit were registered into the cohort upon their consent. If the persons moved out from the clusters, they were censored from the study.

### Malaria diagnosis

All malaria cases in this study were diagnosed by microscopy following the standard malaria diagnosis procedure. During the ACD and MBS, axillary temperature and fever history were collected. Those with fever or reported a history of fever received an on-site malaria testing using a rapid diagnostic test kit (RDT). Malaria cases detected by PCD were diagnosed by microscopy, the routine malaria diagnostic method at the local malaria clinics and hospitals. Microscopic confirmation by experienced microscopists was also performed for all blood samples collected via ACD, MBS, and PCD activities. All confirmed malaria cases were treated according to the national standard guidelines, and were followed in the next weekly home visits.

### Definitions of single or multiple episodes within 1 year

Data on malaria episodes of individuals in the cohort were combined from ACD, MBS, and PCD activities. People in the cohort with no infection detected during ACD, PCD or MBS were considered “not infected”, while those with malaria infections detected only once in the study period were “single episode” cases. For people with multiple malaria episodes, subsequent episodes were defined as those that occurred after 7 days to 1 year from the first episode. This is based on the clinical efficacy of the anti-malarial drugs: *P. falciparum* parasites are normally cleared within 5 days of artemisinin-based combination therapy (ACT) [23], and the parasite clearance time for most *P. vivax* cases treated with the chloroquine-primaquine combination is < 150 h [24]. The recurrent episodes occurred longer than 1 year after the first episode were excluded.

### Risk factors at the individual and household levels

Major risk factors examined here included gender, age, ethnic groups, education, occupational risk, and having other infected person(s) in the same household at the same time. These variables represent potential malaria risk factors among individuals in the area. Age was classified into five different age groups: young child (< 5 years), child (6–10 years), young adult (11–20 years), working age adult (21–60 years), and older person (> 60 years). There were three types of ethnic groups in the study areas: Thai, Karen and others (mostly Myanmar). Occupational risk was classified by characteristics of work environment in the study area and chance of having an infection either as indigenous or imported cases including: low-risk work, child/student, factory labourer, plantation worker, high-risk work and unspecified. Low-risk work included housekeeper/housewife, shopkeeper, monk, government officer, retired and unemployed. Plantation work included those who worked in different types of plantations including cassava, corn, rice and sugar cane in the study areas. High-risk work included hunter, lumberjack/forest worker, orchardist, rubber tree plantation worker, and soldier/police. Having other infected person in the same household at the same time was enumerated among cases residing in the same residential address and the dates of malaria positive test results of the cases were within ± 7 days. Furthermore, risk factors at the household level also included positive cases residing in the same cluster within the same month.

### Statistical analysis

Pattern of malaria infections may be due to variation at individual and household or cluster level [20]. This study analysed individual-level risk factors separately from household-level as well as combined variations of individual-level data within household units. Repeated events in any diseases can be analysed with different statistical models. Some used simple Chi square test for trend while others used models accounting for the correlation between multiple events within subjects [25–27]. Like other malaria studies on counting of repeated events and/or multiple outcomes, this study employed the ordinal logistic model [27, 28]. This model estimates the odds ratio comparing odds of those in the cohort who have never been infected against odds of those who are infected with single episodes and multiple episodes. When the assumption of proportional odds held, the same odds ratio was used to explain the odds of those who had multiple episodes against odds of those who were not infected and who had single infections [29, 30]. In the analysis of time to subsequent episodes, Kaplan–Meier curve was performed.

## Results

### Malaria occurrence during the 6.5-year study period

The study cohort comprised of 7812 people in 1196 houses residing in different sizes of the selected seven clusters ranging from 3315 to 64 people (Table 1). During the 6.5-year period, 410 (5.2%) patients with 527 malaria episodes were observed. Among those, 340 episodes (64.5%) were due to *P. vivax* infection, while 181 (34.3%) and 6 (1.1%) episodes were caused by *P. falciparum* and mixed infections, respectively. Of the 410 malaria cases, 83 patients (20%) had multiple malaria episodes over the study period, ranging from 2 to 5 malaria episodes. About 80% (N = 67) of patients with multiple malaria episodes had the recurrent episodes within 1 year after the primary infection.

**Table 1:** Multiple malaria episodes within 1 year after the initial infection

Clusters	Individual level				Household level (HH)			
	Total population	Malaria cases^a^	Malaria episode^a^	Multiple episode case^b^	Total HH	HH with malaria cases^c^	HH with repeated malaria cases^d^	HH with multiple cases at the same period^d^
	N	n (%)	n (%)	n (%)	N	n (%)	n (%)	n (%)
1	3315	88 (2.7)	98 (3.0)	8 (9.1)	447	70 (15.7)	7 (10.0)	5 (7.1)
2	1902	29 (1.5)	32 (1.7)	3 (10.3)	298	26 (8.7)	3 (11.5)	1 (3.8)
3	1004	139 (13.8)	184 (18.3)	33 (23.7)	190	75 (39.5)	23 (30.7)	12 (16.0)
4	1126	88 (7.8)	96 (8.5)	7 (8.0)	171	58 (33.9)	6 (10.3)	6 (10.3)
5	258	26 (10.1)	30 (11.6)	3 (11.5)	61	19 (31.1)	3 (15.8)	1 (5.3)
6	143	19 (13.3)	28 (19.6)	6 (31.6)	26	11 (42.3)	5 (45.5)	3 (27.3)
7	64	21 (32.8)	31 (48.4)	7 (33.3)	3	3 (100)	3 (100)	3 (100)
Total	7812	410 (5.2)	499 (6.4)	67 (16.3)	1196	262 (21.9)	50 (19.1)	31 (11.8)

^a^Percentage by total population

^b^Percentage by malaria cases

^*c*^Percentage by total household

^d^Percentage by household with malaria cases

### Pattern of multiple episodes of malaria infections within 1 year

Among the 410 malaria patients, 67 (16.3%) had multiple malaria episodes within 1 year. The proportion of multiple episode cases varied across clusters and did not correlate with cluster’s baseline malaria incidence. In Cluster 2 with the lowest malaria incidence level (1.7%), about 10% of malaria cases had multiple malaria episodes, whereas in Cluster 4 with the baseline malaria incidence of 8.5%, the proportion of cases with multiple episodes was observed only in 8%. In Cluster 6 and 7, more than 30% of malaria cases had multiple episodes.

When considering malaria occurrence at the household level, malaria cases were observed in 21.9% of the houses (N = 262) over the 6.5 years (Table 1). Moreover, 31 (11.8%) houses had ≥ 2 family members with malaria infections at the same period. There were only 3 houses in Cluster 7 which had multiple malaria cases. This cluster is a place that fosters orphaned children. In addition, among 262 houses with malaria cases, 50 houses (19.1%) had at least one family member who had multiple malaria episodes within 1 year. If these houses were considered as high-risk houses, the proportion of high-risk houses varied across different clusters. Besides Cluster 7 (orphanage), about 30% and 45% of houses in Cluster 3 and 6 were high-risk houses, respectively. In comparison, only a small proportion of houses (10–15%) in other clusters were high-risk houses.

**Fig. 1:**
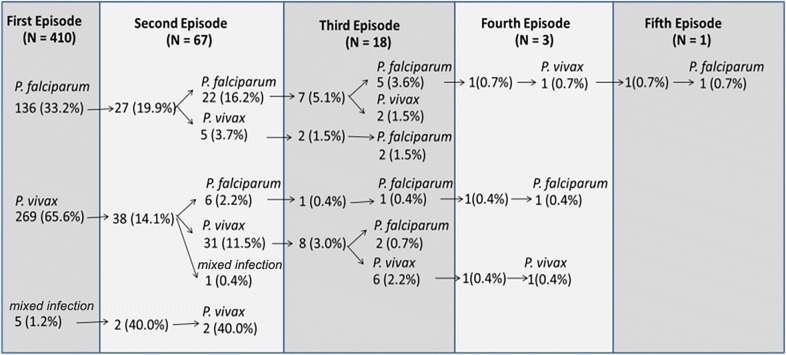
Pattern of repeated malaria episodes within 1 year

For the 67 individuals with multiple episodes of malaria, 73% (N = 49) had two malaria episodes within 1 year. It is noteworthy that one individual had five malaria episodes within 1 year. The majority of the repeated malaria episodes were due to infections by the same *Plasmodium* species (Fig. 1). For instance, among the 27 patients with *P. falciparum* at the first episodes who had subsequent malaria attacks, 22 (81.5%) of the subsequent infections were also due to *P. falciparum*. Similarly, 31/38 (81.6%) patients with *P. vivax* at the first episode also had *P. vivax* at the recurrent episodes. Mixed infection (*P. falciparum* and *P. vivax)* was observed in five cases, two of which had repeated episodes with *P. vivax*.

### Time to subsequent episodes within 1 year

**Fig. 2:**
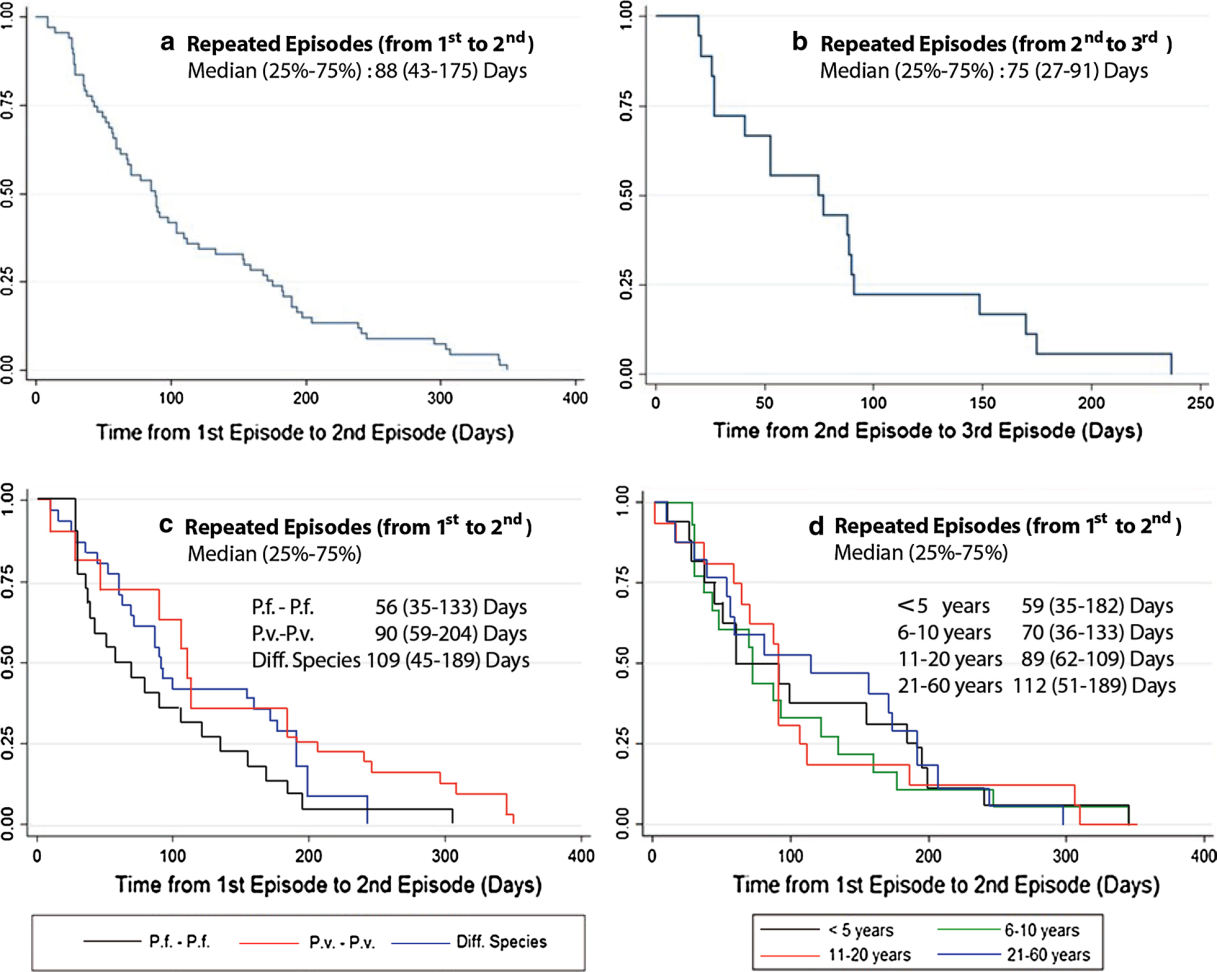
Interval (days) between repeated episodes within 1 year

As shown in Fig. 2, the median time from the first to the second episode was 88 days with interquartile range (IQR) of 43–175 days. The median time from the second to the third episode was relatively shorter at 75 days with IQR of 27–91 days. When exploring time from the first to the second episode among those who were infected at the second time with the same parasite species, two repeated *P. falciparum* infections showed the shortest interval with a median of 56 (IQR: 35–133) days. For two repeated *P. vivax* episodes, the median time was 90 (IQR: 59–204) days. A longer interval was observed among those who were infected with different parasite species—the median time was 109 (IQR: 45–189) days. Trend in time from the first to the second episode was shown when exploring different age groups. For patients aged < 5 years, the median time was 59 (IQR: 35–182) days, whereas for those aged 6–10 years the median time was 70 (IQR: 36–183) days. For patients aged 11–20 and 21–60 years, the median time was 89 (IQR: 62–109) and 112 (IQR: 51–189) days, respectively.

### Factors associated with repeated malaria episodes

Different statistically significant risk factors for repeated malaria attacks were identified (Table 2). Males had higher odds of having multiple episodes than females (OR: 1.46; 95% CI 1.19–1.78). Compared to children aged < 5 years, those age 6–11 years had higher odds (OR: 1.74; 95% CI 1.30–2.33). Adults at working age group of 21–60 had lower odds of having multiple episodes compared to children age < 5 years (OR: 0.68; 95% CI 0.51–0.89); and no older adults had multiple episodes (OR: 0.20; 95% CI 0.11–0.65). The Karen ethnic group had higher odds than the local Thai (OR: 1.38; 95% CI 1.02–1.88). Those with some levels of education had lower odds of having multiple episodes when compared to those who were illiterate; however, only education at secondary level showed a significant association. For occupation, when compared with those in the low-risk work category, students and children had the highest odds ratio (OR: 3.95; 95% CI 2.30–6.81) followed by unspecified (other) occupation (OR: 3.74; 95% CI 2.06–6.78), factory workers (OR: 3.70; 95% CI 1.91–7.16), and plantation worker (OR: 2.00; 95% CI 1.09–3.68). The odds of having multiple episodes was not significantly different between those working in high-risk and low-risk work categories. However, a relatively small number of people were in the high-risk category. Having another infected person in the same household at the same period conferred a higher risk to have multiple episodes comparing to a single episode (OR: 3.05; 95% CI 1.71–5.45).

**Table 2:** Risk factor analysis of multiple episodes of malaria in western Thailand

Characteristics	Total	Not infected	Single episode	Multiple episodes	Odds ratio^a^ (95% CI)
	N	n (%)	n (%)	n (%)	
Sex					
Female	3908	3739 (95.7)	145 (3.7)	24 (0.6)	1
Male	3904	3663 (93.8)	198 (5.1)	43 (1.1)	1.46 (1.19–1.78)
Age groups					
< 5	1755	1657 (94.4)	82 (4.7)	16 (0.9)	1
6–10	1051	953 (90.7)	80 (7.6)	18 (1.7)	1.74 (1.30–2.33)
11–20	1705	1610 (94.4)	79 (4.7)	16 (0.9)	1.00 (0.75–1.33)
21–60	2963	2849 (96.1)	97 (3.3)	17 (0.6)	0.68 (0.51–0.89)
> 60	328	323 (98.5)	5 (1.5)	0 (0.0)	0.26 (0.11–0.65)
Ethnic groups					
Thai	1083	1034 (95.5)	47 (4.3)	2 (0.2)	1
Karen	5703	5355 (93.9)	284 (5.0)	64 (1.1)	1.38 (1.02–1.88)
Other	51	50 (98.0)	1 (2.0)	0 (0.0)	0.42 (0.06–3.13)
Education attainment					
Illiterate	4280	4035 (94.3)	205 (4.8)	40 (0.9)	1
Primary	1700	1580 (92.9)	98 (5.8)	22 (1.3)	1.25 (0.99–1.57)
Secondary	824	793 (96.2)	28 (3.4)	3 (0.4)	0.64 (0.44–0.94)
College	19	18 (94.7)	0 (0.0)	1 (5.3)	0.96 (0.13–7.23)
Occupational risk					
Low risk work	742	728 (98.1)	13 (1.8)	1 (0.1)	1
Child/student	3632	3376 (92.9)	210 (5.8)	46 (1.3)	3.95 (2.30–6.81)
Factory laborers	393	367 (93.4)	21 (5.3)	5 (1.3)	3.70 (1.91–7.16)
Plantation workers	1187	1143 (96.3)	38 (3.2)	6 (0.5)	2.00 (1.09–3.68)
High risk work	55	53 (96.4)	2 (3.6)	0 (0.0)	1.95 (0.43–8.82)
Unspecified	820	765 (93.2)	47 (5.8)	8 (1.0)	3.74 (2.06–6.78)
Having other infected person in the same HH^b^					
No	333	–	290 (87.1)	43 (12.9)	1
Yes	77	–	53 (66.8)	24 (31.2)	3.05 (1.71–5.45)

^a^Odds ratios were based on proportional ordinal logistic model

^b^Odds ratio was based on binary logistic model (comparing only infected cases)

## Discussion

Recurrent malaria infection could have an impact on both individual’s health and malaria transmission in the community. Even in malaria low-transmission areas, recurrent parasitaemia after initial treatment is still observed. In this area along the Thai-Myanmar border, the incidence of malaria occurred in 5.2% of the studied cohort over 6.5-year period. Yet, ~ 20% of malaria cases still had recurrent episodes; most of recurrent episodes occurred within 1 year after the initial infection. The maximum number of malaria episodes an individual had within 1 year was 5 times. Previous studies conducted in high malaria transmission areas in Africa reported that malaria episodes among children ranged from 0 to 40 per child over a 5-year period [4, 31]. Even in a low-transmission area in Africa, children could experience multiple episodes ranging from 5 to 16 times during a 5-year follow-up [4]. Although multiple malaria episodes may be the result of increased malaria exposure, the scenario encountered here deserves further analysis, as the epidemiological settings including baseline malaria incidence, predominant *Plasmodium* spp, vectors, and environment, vary considerably.

Recurrent malaria episodes may be due to recrudescence, relapses, or new infections. This study, however, did not differentiate the causes of these recurrent episodes. Yet, in low-malaria transmission areas, the annual incidence of new infections is typically low and estimated to be less than one per person [32]. This suggests that the probability of reinfection with new mosquito bites within 1 year is low in low-endemic settings such as in western Thailand. In this study, 80% of patients with multiple malaria episodes had recurrent episodes occurring within 1 year after the initial infection, and most of the recurrent episodes were infected with the same malaria species as the primary infection. It is thus reasonable to speculate that most of these recurring infections were due to either recrudescence or relapse.

Inadequate anti-malarial treatment and drug resistance may result in recrudescence and relapse. Recrudescence is caused by the remaining blood-stage parasites, which failed to be completely eliminated by anti-malarial treatment. The GMS is known as an epicenter of multidrug-resistant *P. falciparum* parasites. Per the NMCP anti-malarial drug policy, falciparum cases were treated by ACT (in this case artesunate-mefloquine: AS-MQ). However, due to the emergence and spread of artemisinin-resistant *P. falciparum* parasites in the GMS [33], treatment efficacy of this ACT has been compromised. A previous study in western Cambodia along the Thai-Cambodia border reported 21% failure rate of AS-MQ treatment within 42 days after the initial treatment [33]. In this study, about 25% of recurrent episodes among patients primarily infected with *P. falciparum* followed by another *P. falciparum* episode occurred within 35 days, highlighting the possibility of recrudescence of falciparum malaria due to drug resistance. For vivax malaria, the standard treatment chloroquine-primaquine (CQ-PQ) for uncomplicated *P. vivax* infection remained generally effective in the GMS, although sporadic CQ-resistant *P. vivax* parasites have been reported in this region [34]. In this study, 15% of recurrent episodes among patients with primary and recurrent infections by *P. vivax* occurred within 35 days, which also suggests deteriorating CQ efficacy in this region. This demands close monitoring of anti-malarial drug resistance in this area.

Relapse is a result of hypnozoite reactivation during *P. vivax* and *P. ovale* infections. Duration of relapses varies by the parasite strains; in the tropical region of Southeast Asia, *P. vivax* strains, such as the Chesson strain, relapse within 1–5 months [18]. A study conducted in Brazil showed that relapse episodes mostly occurred between the day 28 and 90 after initiating treatment [9]. Similarly in Colombia, 86% of the first *P. vivax* recurrent events occurred between day 51 and 110, and 65.5% of these subsequent episodes were genetically classified as relapses [7]. Identification of relapse is challenging as the relapse episodes could be caused by the activation of the parasites that have different genotypes from that of the initial infection [35, 36]. In this study, the median time interval between the first and second *P. vivax* infection was 90 days, which are likely due to relapse. In addition, this study also identified that 3.7% of *P. falciparum* primary infection had subsequent episode of *P. vivax* infections*. Plasmodium vivax* recurrence after treatment of primary *P. falciparum* infection has been reported in previous studies conducted along the Thai-Myanmar border [37]. Since AS-MQ was presumably effective for clearing blood-stage *P. vivax* infections, the subsequent episodes of vivax infection after *P. falciparum* treatment are also likely due to relapses. Adequate PQ treatment can substantially reduce the risk of relapses [38]. In the study area, the 14-day of PQ regimen (15 mg) is used for radical cure of vivax infections. However, the main challenge for PQ treatment is patient’s compliance to the 14-day regimen. Previous study conducted along the Thai-Myanmar border reported that about 15% of vivax malaria patients had missed at least one dose of PQ; and poor compliance was associated with an increased risk of *P. vivax* recurrence [39]. Therefore, better case management of vivax malaria will help reduce recurrence of the disease.

Many factors have been associated with malaria infections [40–44]. This study identified the male sex, young age, Karen ethnic, certain high-risk occupations, and concurrently having other infected persons in the same household as the significant risk factors for having recurrent malaria episodes. Males had higher odds than females in multiple infections, compared to single episode and malaria-free cases. Previous studies showed inconsistent results on being male as a risk factor for malaria infection [3–5, 45]. This could be due to difference in malaria protection behaviours and occupation among males across different areas [5, 46]. In this area, males usually engage in outdoor works. Some people may have agricultural plantations far from their houses; they may need to stay in temporary shelters at the plantations [47]. This could increase the risk of mosquito exposure. In addition, a study conducted in another area along the Thai-Myanmar border also suggested that males were more likely to not adhere to the anti-malarial drug treatment regimen than females [39]. This may increase the risk of recrudescence or relapse.

Like what has been found in earlier studies [4, 8], children were at a higher risk of malaria recurrence. Whereas this could be due to many factors, inadequate anti-malarial drug dosing may be partially responsible. In most endemic settings, anti-malarial drug dosage is defined by different age groups, rather than by the body weight of the child patient. Therefore, children were more likely to receive under-dosed anti-malarial treatment [38]. By examining the duration between the first and second episode of malaria among different age groups, it appeared that the median time for young children was shorter than those of older ages. This implies that the shorter duration of the recurrent episodes (recrudescence or relapse) in children may likely be due to inadequate treatment.

The majority of patients in this study belongs to the Karen ethnic group, one of the largest ethnic minority groups along the Thai-Myanmar border. Karen people mainly work as workers in local factories or in the plantations/orchards near the border areas. Among those who work in the agricultural settings, some maintain farming plots on the Myanmar side of the border, whereas some might reside in agricultural huts only during peak harvesting times of the year [48]. Such practices contribute to occupational risk which was also found in this study as a significant risk factor of malaria infection and multiple malaria episodes. Migration across international borders has been discussed widely in literature as a major risk factor of malaria infection as many of them work in high-risk environments such as natural forest [8, 48]. Although not many people in this cohort reported to have worked in the forest and most Karen and other ethnic groups (mostly Myanmar) tend to reside in stable settings in Thailand, they may frequently cross international border to visit weekly markets or stay overnight with relatives and friends.

Having other infected person in the same household at the same period was a significant risk factor for having multiple malaria episodes in this cohort study. In areas where *Anopheles* vectors are abundant and effective in malaria transmission [49, 50], human reservoir could play an important role in local malaria transmission in the area [51]. In this study area, *Anopheles minimus* and *Anopheles maculatus* are the main malaria vectors. Previous study has shown that monthly *Anopheles* capture rate was associated with *P. vivax* monthly incidence, suggesting a potential role of these vectors in local malaria transmission [52]. A socio-economic study conducted in two clusters of the study area showed that approximately 80% houses were made from bamboo and wood, with elevated floor structure. In addition, about 87% of houses had household monthly income < 5000 Thai Baht. Almost 80% of houses own bed nets. These household characteristics were associated with self-reported malaria history [47]. However, further study is needed to confirm whether these factors are associated with multiple malaria episodes among household members. In addition, a spatial analysis on these high-risk houses, including entomological factors and proximity to high-risk houses and mosquito breeding sites, will provide better understanding on the malaria transmission pattern in the area.

In this study, there was no information available to differentiate between recrudescence, relapse, and re-infection. Molecular studies may allow us to identify the types of recurrence. In addition, this study did not cover the issue of drug resistance in the study area that might be related to transmission intensity, multiplicity of infections, and long-term persistence of resistant parasites [53]. Moreover, the analysis of recurrent episodes in this study did not include the parasitaemia occurred within 7 days after the initial treatment. Nevertheless, this study identified that multiple malaria episodes were associated with asymptomatic or subclinical infections even in low-transmission areas. Multiple cross-sectional MBS were conducted every 4–6 months during 2011–2015, which aimed to detect asymptomatic parasitaemic cases. Although this is resource-demanding, MBS to survey asymptomatic or submicroscopic parasitaemia is quite informative on parasite prevalence and should be performed periodically. Understanding the distribution pattern and risk factors of those submicroscopic parasitaemias is considered one of the key issues in moving toward elimination [54]. The analyses on asymptomatic and submicroscopic parasitaemias will be further studied intensively in this malaria pre-elimination area.

## Conclusion

In a low-transmission area where the cumulative malaria incidence was only 5.2% over the 6.5-year period, 16% of all infected patients were still found to have multiple malaria episodes within 1 year. Children, male, Karen ethnicity, working in forest-related occupation, and living currently in the same house with malaria case were significant risk factors associated with multiple malaria episodes. These people with multiple malaria episodes could serve as potential human reservoirs that require special attention, particularly during the malaria pre-elimination phase. Although the causes of the recurrent episodes were not genetically identified, inadequate malaria treatment either due to anti-malarial drug resistance or poor drug compliance should be closely monitored to prevent recrudescence and relapses. Understanding of the local epidemiology in terms of distinguishing locally acquired infection versus cross-border infection is important to guide successful interventions in moving toward malaria elimination. Further intensive studies are needed on adaptive strategies for different areas to achieve the ultimate elimination goal.

## Abbreviations

95% CI: 95% confidence interval
ACD: active case detection
GMS: Greater Mekong Subregion
MBS: mass blood survey
IQR: interquartile range, between 25 and 75%
PCD: passive case detection

## References

[1] Rogier C, Tall A, Diagne N, Fontenille D, Spiegel A, Trape JF (1999). *Plasmodium falciparum* clinical malaria: lessons from longitudinal studies in Senegal. Parassitologia.

[2] Rogier C, Imbert P, Tall A, Sokhna C, Spiegel A, Trape JF (2003). Epidemiological and clinical aspects of blackwater fever among African children suffering frequent malaria attacks. Trans R Soc Trop Med Hyg.

[3] Ndungu FM, Marsh K, Fegan G, Wambua J, Nyangweso G, Ogada E (2015). Identifying children with excess malaria episodes after adjusting for variation in exposure: identification from a longitudinal study using statistical count models. BMC Med.

[4] Rono J, Farnert A, Murungi L, Ojal J, Kamuyu G, Guleid F (2015). Multiple clinical episodes of *Plasmodium falciparum* malaria in a low transmission intensity setting: exposure versus immunity. BMC Med.

[5] Douglas NM, Nosten F, Ashley EA, Phaiphun L, van Vugt M, Singhasivanon P (2011). *Plasmodium vivax* recurrence following falciparum and mixed species malaria: risk factors and effect of antimalarial kinetics. Clin Infect Dis.

[6] Ndiaye JL, Faye B, Gueye A, Tine R, Ndiaye D, Tchania C (2011). Repeated treatment of recurrent uncomplicated *Plasmodium falciparum* malaria in Senegal with fixed-dose artesunate plus amodiaquine versus fixed-dose artemether plus lumefantrine: a randomized, open-label trial. Malar J.

[7] Zuluaga-Idarraga L, Blair S, Akinyi Okoth S, Udhayakumar V, Marcet PL, Escalante AA (2016). Prospective study of *Plasmodium vivax* malaria recurrence after radical treatment with a chloroquine-primaquine standard regimen in Turbo, Colombia. Antimicrob Agents Chemother.

[8] Howes RE, Battle KE, Mendis KN, Smith DL, Cibulskis RE, Baird JK (2016). Global epidemiology of *Plasmodium vivax*. Am J Trop Med Hyg.

[9] Simoes LR, Alves ER, Ribatski-Silva D, Gomes LT, Nery AF, Fontes CJ (2014). Factors associated with recurrent *Plasmodium vivax* malaria in Porto Velho, Rondonia State, Brazil, 2009. Cad Saude Publica.

[10] WHO (2015). Guidelines for the treatment of malaria.

[11] Baird JK (2013). Malaria caused by *Plasmodium vivax*: recurrent, difficult to treat, disabling, and threatening to life—the infectious bite preempts these hazards. Pathog Glob Health.

[12] Chaves YO, da Costa AG, Pereira ML, de Lacerda MV, Coelho-Dos-Reis JG, Martins-Filho OA (2016). Immune response pattern in recurrent *Plasmodium vivax* malaria. Malar J.

[13] Fernando SD, Gunawardena DM, Bandara MR, De Silva D, Carter R, Mendis KN (2003). The impact of repeated malaria attacks on the school performance of children. Am J Trop Med Hyg.

[14] Vorasan N, Pan-Ngum W, Jittamala P, Maneeboonyang W, Rukmanee P, Lawpoolsri S (2015). Long-term impact of childhood malaria infection on school performance among school children in a malaria endemic area along the Thai-Myanmar border. Malar J.

[15] Seyoum D, Kifle YG, Rondeau V, Yewhalaw D, Duchateau L, Rosas-Aguirre A (2016). Identification of different malaria patterns due to *Plasmodium falciparum* and *Plasmodium vivax* in Ethiopian children: a prospective cohort study. Malar J.

[16] Parker DM, Matthews SA, Yan G, Zhou G, Lee MC, Sirichaisinthop J (2015). Microgeography and molecular epidemiology of malaria at the Thailand-Myanmar border in the malaria pre-elimination phase. Malar J.

[17] Carrara VI, Lwin KM, Phyo AP, Ashley E, Wiladphaingern J, Sriprawat K (2013). Malaria burden and artemisinin resistance in the mobile and migrant population on the Thai-Myanmar border, 1999–2011: an observational study. PLoS Med.

[18] White NJ (2011). Determinants of relapse periodicity in *Plasmodium vivax* malaria. Malar J.

[19] Bannister-Tyrrell M, Verdonck K, Hausmann-Muela S, Gryseels C, Muela Ribera J, Peeters Grietens K (2017). Defining micro-epidemiology for malaria elimination: systematic review and meta-analysis. Malar J.

[20] Department for International Development (DFID) Malaria: Burden and Interventions, Evidence Overview. A working paper (version 1.0). https://assets.publishing.service.gov.uk/media/57a08af5ed915d3cfd000a46/malaria-evidence-paper.pdf. Accessed 15 Oct 2018.

[21] WHO (2018). World malaria report 2017.

[22] Moss WJ, Dorsey G, Mueller I, Laufer MK, Krogstad DJ, Vinetz JM (2015). Malaria epidemiology and control within the International Centers of Excellence for Malaria Research. Am J Trop Med Hyg.

[23] Dondorp AM, Fairhurst RM, Slutsker L, Macarthur JR, Breman JG, Guerin PJ (2011). The threat of artemisinin-resistant malaria. N Engl J Med.

[24] White NJ (2017). Malaria parasite clearance. Malar J.

[25] WorldWide Antimalarial Resistance Network (WWARN). Statistical Analysis Plan, WWARN Vivax Recurrence Study Group: a pooled analysis investigating the effect of mg/kg drug dosage on *Plasmodium vivax* recurrence, version 0.1. http://www.wwarn.org/sites/default/files/attachments/documents/wwarn_sap_recurrence_290117.pdf. Accessed 15 Oct 2018.

[26] Macera MA, Louzada F, Cancho VG, Fontes CJ (2015). The exponential-Poisson model for recurrent event data: an application to a set of data on malaria in Brazil. Biom J.

[27] Khaireh BA, Briolant S, Pascual A, Mokrane M, Machault V, Travaille C (2012). *Plasmodium vivax* and *Plasmodium falciparum* infections in the Republic of Djibouti: evaluation of their prevalence and potential determinants. Malar J.

[28] WWARN Artemisinin based Combination Therapy (ACT) Africa Baseline Study Group (2015). Clinical determinants of early parasitological response to ACTs in African patients with uncomplicated falciparum malaria: a literature review and meta-analysis of individual patient data. BMC Med.

[29] Williams R (2016). Understanding and interpreting generalized ordered logit models. J Math Sociol.

[30] Institute for Digital Research and Education, UCLA. Ordered logistic regression. https://stats.idre.ucla.edu/stata/dae/ordered-logistic-regression/. Accessed 1 Mar 2018.

[31] Mwangi TW, Fegan G, Williams TN, Kinyanjui SM, Snow RW, Marsh K (2008). Evidence for over-dispersion in the distribution of clinical malaria episodes in children. PLoS ONE.

[32] Yukich J, Briet O, Bretscher MT, Bennett A, Lemma S, Berhane Y (2012). Estimating *Plasmodium falciparum* transmission rates in low-endemic settings using a combination of community prevalence and health facility data. PLoS ONE.

[33] Woodrow CJ, White NJ (2017). The clinical impact of artemisinin resistance in Southeast Asia and the potential for future spread. FEMS Microbiol Rev.

[34] Baird JK (2009). Resistance to therapies for infection by *Plasmodium vivax*. Clin Microbiol Rev.

[35] Imwong M, Snounou G, Pukrittayakamee S, Tanomsing N, Kim JR, Nandy A (2007). Relapses of *Plasmodium vivax* infection usually result from activation of heterologous hypnozoites. J Infect Dis.

[36] Chen N, Auliff A, Rieckmann K, Gatton M, Cheng Q (2007). Relapses of *Plasmodium vivax* infection result from clonal hypnozoites activated at predetermined intervals. J Infect Dis.

[37] Commons RJ, Simpson JA, Thriemer K, Hossain MS, Douglas NM, Humphreys GS (2019). Risk of *Plasmodium vivax* parasitaemia after *Plasmodium falciparum* infection: a systematic review and meta-analysis. Lancet Infect Dis.

[38] Commons RJ, Simpson JA, Thriemer K, Humphreys GS, Abreha T, Alemu SG (2018). The effect of chloroquine dose and primaquine on *Plasmodium vivax* recurrence: a WorldWide Antimalarial Resistance Network systematic review and individual patient pooled meta-analysis. Lancet Infect Dis.

[39] Takeuchi R, Lawpoolsri S, Imwong M, Kobayashi J, Kaewkungwal J, Pukrittayakamee S (2010). Directly-observed therapy (DOT) for the radical 14-day primaquine treatment of *Plasmodium vivax* malaria on the Thai-Myanmar border. Malar J.

[40] Clark TD, Greenhouse B, Njama-Meya D, Nzarubara B, Maiteki-Sebuguzi C, Staedke SG (2008). Factors determining the heterogeneity of malaria incidence in children in Kampala, Uganda. J Infect Dis.

[41] Kreuels B, Kobbe R, Adjei S, Kreuzberg C, von Reden C, Bater K (2008). Spatial variation of malaria incidence in young children from a geographically homogeneous area with high endemicity. J Infect Dis.

[42] Trape JF, Lefebvre-Zante E, Legros F, Ndiaye G, Bouganali H, Druilhe P (1992). Vector density gradients and the epidemiology of urban malaria in Dakar, Senegal. Am J Trop Med Hyg.

[43] Midega JT, Smith DL, Olotu A, Mwangangi JM, Nzovu JG, Wambua J (2012). Wind direction and proximity to larval sites determines malaria risk in Kilifi District in Kenya. Nat Commun.

[44] Bousema T, Drakeley C, Gesase S, Hashim R, Magesa S, Mosha F (2010). Identification of hot spots of malaria transmission for targeted malaria control. J Infect Dis.

[45] Parker DM, Tripura R, Peto TJ, Maude RJ, Nguon C, Chalk J (2017). A multi-level spatial analysis of clinical malaria and subclinical Plasmodium infections in Pailin Province, Cambodia. Heliyon.

[46] Olapeju B, Choiriyyah I, Lynch M, Acosta A, Blaufuss S, Filemyr E (2018). Age and gender trends in insecticide-treated net use in sub-Saharan Africa: a multi-country analysis. Malar J.

[47] Saita S, Pan-Ngum W, Phuanukoonnon S, Sriwichai P, Silawan T, White LJ, Parker DM (2019). Human population movement and behavioural patterns in malaria hotspots on the Thai-Myanmar border: implications for malaria elimination. Malar J.

[48] Parker DM, Wood JW, Tomita S, DeWitte S, Jennings J, Cui L (2014). Household ecology and out-migration among ethnic Karen along the Thai-Myanmar border. Demogr Res.

[49] Poolphol P, Harbach RE, Sriwichai P, Aupalee K, Sattabongkot J, Kumpitak C (2017). Natural *Plasmodium vivax* infections in Anopheles mosquitoes in a malaria endemic area of northeastern Thailand. Parasitol Res.

[50] Sriwichai P, Samung Y, Sumruayphol S, Kiattibutr K, Kumpitak C, Payakkapol A (2016). Natural human Plasmodium infections in major Anopheles mosquitoes in western Thailand. Parasit Vectors.

[51] Lawpoolsri S, Chavez IF, Yimsamran S, Puangsa-Art S, Thanyavanich N, Maneeboonyang W (2010). The impact of human reservoir of malaria at a community-level on individual malaria occurrence in a low malaria transmission setting along the Thai-Myanmar border. Malar J.

[52] Sriwichai P, Karl S, Samung Y, Kiattibutr K, Sirichaisinthop J, Mueller I (2017). Imported *Plasmodium falciparum* and locally *transmitted Plasmodium vivax*: cross-border malaria transmission scenario in northwestern Thailand. Malar J.

[53] Parker DM, Carrara VI, Pukrittayakamee S, McGready R, Nosten FH (2015). Malaria ecology along the Thailand-Myanmar border. Malar J.

[54] Lin JT, Saunders DL, Meshnick SR (2014). The role of submicroscopic parasitemia in malaria transmission: what is the evidence?. Trends Parasitol.

